# Vocation or Living

**Published:** 1907-05-18

**Authors:** 


### Nursing Administration.

### VOCATION OR LIVING.

The distinction between the woman to whom nursing is a vocation and the woman who merely earns her living by nursing, is a very real one, although it is a very difficult one to define. It does not lie in the possession of any particular quality by the one which is lacking to the other. A nurse may be full of faults and yet have a true vocation; while, on the other hand, a woman of the most estimable type may be lacking in the smallest spark of the divine fire. Nor does vocation lie in outward circumstances of birth, or education, or wealth. The vocation for nursing leaps out from the humblest, as well as from the highest homes in the kingdom. Its one shining mark is its vitality. It is able to stand a test. The nurse with a vocation may fail miserably in her first month's trial, and even be dismissed with well-meant advice to try other work. Undaunted she will begin lower down, and after a year in a smaller institution, will try again and succeed. The woman with a vocation may be hampered by external circumstances, as was one of the ablest matrons among us, on whom the task was laid of mothering twelve brothers and sisters through their school days, before her life work could begin. She may be naturally bad-tempered and impatient of control. A true vocation will teach her self-command. She may be rich and pleasure loving. Her vocation will lead her to love a simple life. She may be ignorant. Her vocation will be to her an education. It is only through conscientious surrender to training that the vocation can be perfected, and it is equally true that only in those who possess vocation can training do its highest work.

But there is much to be said for the simple straightforward motive of desiring to earn a living. It is an unsafe thing for any profession to depend for its supply of members on exceptional people. And women with a purpose in life strong enough to overbalance all personal considerations, are exceptional. There are many safeguards in the necessity for looking to the work of one's own hands for independence. The woman who sets out to earn her own living knows that every step in her career will either advance or retard her future prospects, and the knowledge works tolerance under unpleasant conditions, patience under apparent injustice, simplicity in the discharge of mechanical duties, in short, a spirit of duty. The labourer is worthy of his hire. And no strong development of nursing can be anticipated in directions which do not afford a prospect of independence and the means of securing a future competence. It is for this reason that the development of village nursing is at the present moment grievously cramped, notwithstanding the urgent call for workers in this field.

When the hospital training is ended, and the nurse looks out into the future, the problem of "vocation" or "living" becomes an insistent one. It may be broadly laid down that "vocation" trends more to the nursing of the sick poor in hospitals, and in district work; while "living" is best secured in private nursing among the rich. It would be ridiculous to claim a spirit of pure devotion to their calling for all nurses working in institutions, just as it would be impertinent to deny it to those working in the homes of the rich. Many women cling to institution life from a preference for routine or want of energy to make a change long after a change would be desirable from all points of view. And many women are carrying out their vocation in the highest sense of the term, under the varied and difficult conditions which surround the private nurse. But it is necessary to face the fact that for the woman who enters the calling of the nurse, simply and solely to make money, there are attractions in private nursing which are not to be found in any other branch of nursing work. The unfortunate consequence of this is that the defects of these nurses—they are often exceedingly clever from a technical point of view—are displayed just where they are thrown into most prominence. Probably this fact accounts in great measure for the extraordinary virulence exhibited occasionally towards nurses as a class, by persons who ought to know better. It helps to explain why the nurse of fiction, the nurse of the newspaper, is a being unknown to many who yet have passed their lives among nurses. The remedy does not lie in registration, for love of money could scarcely be held by the most drastic disciplinarian a proper ground for removal from a list of registered nurses. The remedy lies in deepening the sense of vocation in nurses wherever their work may lead them, and in instilling into probationers during the time when they are wholly under the influence of the training school, a purpose in life higher than that of the bread winner.

It is difficult to seize and put into words the form under which vocation makes itself felt to the mind. But in its widest developments it is pre-eminently Christian and altruistic. It is the vital principle which desires to give out of the abundance of the heart to those who lack. But as centring on nursing it is the desire for the succour of the body, even more than the mind or the soul, or the outward circumstances. It may be claimed for it in this connection also that it is an eminently Christ-like purpose. So far as God revealed Himself in His Son, He stands revealed as hating sickness and infirmity; willing the race to be strong, healthy, and free from bodily defect. The unhappy view of life which linked "God's will" with disease finds no support in the Gospels, and under the eye of science is rapidly disappearing. The essence of the nurse's vocation is that it calls her forth to aid in that mighty battle against disease in which the banners of God's Will are waving the race on to victory. To-day, for the first time in the history of ages, the foe is losing ground. Not till the citadels of disease and despair have crumbled to the ground shall the nurse's vocation pass from among the inspiring forces of womanhood.

